# Disease-modifying antirheumatic drugs are associated with a reduced risk for cardiovascular disease in patients with rheumatoid arthritis: a case control study

**DOI:** 10.1186/ar2045

**Published:** 2006-09-20

**Authors:** Vokko P van Halm, Michael T Nurmohamed, Jos WR Twisk, Ben AC Dijkmans, Alexandre E Voskuyl

**Affiliations:** 1 Department of Rheumatology, VU University Medical Center, Boelelaan 1117, Amsterdam, 1085 HV, The Netherlands; 2 Department of Rheumatology, Jan van Breemen Institute, Jan van Breemen straat 2, Amsterdam, 1056 AB, The Netherlands; 3 Department of Clinical Epidemiology and Biostatistics, VU University Medical Center, Boelelaan 1117, Amsterdam, 1085 HV, The Netherlands

## Abstract

Rheumatoid arthritis (RA) is characterized by inflammation and an increased risk for cardiovascular disease (CVD). This study investigates possible associations between CVD and the use of conventional disease-modifying antirheumatic drugs (DMARDs) in RA. Using a case control design, 613 RA patients (5,649 patient-years) were studied, 72 with CVD and 541 without CVD. Data on RA, CVD and drug treatment were evaluated from time of RA diagnosis up to the first cardiovascular event or the end of the follow-up period. The dataset was categorized according to DMARD use: sulfasalazine (SSZ), hydroxychloroquine (HCQ) or methotrexate (MTX). Odds ratios (ORs) for CVD, corrected for age, gender, smoking and RA duration, were calculated per DMARD group. Patients who never used SSZ, HCQ or MTX were used as a reference group. MTX treatment was associated with a significant CVD risk reduction, with ORs (95% CI): 'MTX only', 0.16 (0.04 to 0.66); 'MTX and SSZ ever', 0.20 (0.08 to 0.51); and 'MTX, SSZ and HCQ ever', 0.20 (0.08 to 0.54). The risk reductions remained significant after additional correction for the presence of rheumatoid factor and erosions. After correction for hypertension, diabetes and hypercholesterolemia, 'MTX or SSZ ever' and 'MTX, SSZ and HCQ ever' showed significant CVD risk reduction. Rheumatoid factor positivity and erosions both increased CVD risk, with ORs of 2.04 (1.02 to 4.07) and 2.36 (0.92 to 6.08), respectively. MTX and, to a lesser extent, SSZ were associated with significantly lower CVD risk compared to RA patients who never used SSZ, HCQ or MTX. We hypothesize that DMARD use, in particular MTX use, results in powerful suppression of inflammation, thereby reducing the development of atherosclerosis and subsequently clinically overt CVD.

## Introduction

Cardiovascular diseases (CVDs) are the most important cause of death in patients with rheumatoid arthritis (RA). RA is associated with a significant increase in cardiovascular morbidity and mortality compared to the general population [1-7]. A clear explanation for this excess in cardiovascular risk is lacking, although several causes have been postulated. First, an increased prevalence of established cardiovascular risk factors, such as hypertension, diabetes and hypercholesterolemia. Second is the possibility of under-treatment of cardiovascular co-morbidity [8,9]. Third, RA itself could be responsible for the excess in cardiovascular morbidity and mortality, either by a decreased functional capacity [10], or by the underlying inflammatory process. There is growing evidence that atherosclerosis is an inflammatory disease [11,12]. Moreover, inflammation might cause deterioration of fatty streaks into (unstable) plaques [13] and can lead to plaque ruptures [14], as well as to complement activation [15] or facilitate deterioration of the lipid profile [16], all important aspects in the pathogenesis of atherosclerosis.

A complex element in the association between RA and cardiovascular risk is the use of antirheumatic medication. Patients with persisting disease activity require treatment with disease-modifying antirheumatic drugs (DMARDs) [17]. There are some indications that DMARDs can alter cardiovascular risk either by influencing atherosclerotic processes directly through inflammation or indirectly by influencing cardiovascular risk factors [18,19]. However, reports on the relationship between DMARDs and CVD are limited, focused on mortality and predominantly on methotrexate (MTX), and the results are contradictory [20,21]. Therefore, the present study investigates associations between cardiovascular morbidity and the use of several conventional DMARDs.

## Materials and methods

### Patients

Demographic, RA and CVD related data were collected from 613 RA patients by chart review. This sample of RA patients was randomly taken from the entire RA population registered in the Jan van Breemen Institute, a large rheumatology outpatient clinic in Amsterdam, the Netherlands. All patients fulfilled the American College of Rheumatology criteria of RA. Patients were recruited from time of diagnosis, between 1953 and 2002, onwards until March 2004, the end of the follow up period.

### Study design

A case control study of incident CVD was performed, comparing 72 patients with RA and CVD to 541 RA patients without CVD. CVD was evaluated from the time of diagnosis of the RA up to the time of the first cardiovascular event or until the end of the follow up period.

### Cardiovascular disease and risk factors for it

CVD was defined as a verified medical history of coronary, cerebral or peripheral arterial disease. Coronary artery disease included a history of myocardial infarction, a coronary artery by-pass graft procedure, a percutaneous transluminal coronary angioplasty or ischemic abnormalities on ECG. Cerebral arterial disease was defined as a history of cerebral vascular accident (confirmed by a neurologist), a transient ischemic attack or a carotid endarterectomy. Peripheral arterial disease included an aneurysm of the thoracic and/or abdominalis aorta, a peripheral arterial by-pass operation and amputation of the (lower) leg. Assessed risk factors for CVD were age, male sex, hypertension, diabetes, hypercholesterolemia, and smoking habits. Hypertension, diabetes and hypercholesterolemia were considered to be present if patients received treatment for these conditions. Smoking habits were recorded as use ever versus never. All these variables were monitored throughout the entire disease duration.

### Statistical analyses

Comparisons between the various DMARD groups and between the RA patients with CVD and without CVD were performed using Students' t-tests and Mann-Whitney U-tests for continuous variables and Pearson's Chi-square tests for dichotomic variables.

The dataset was categorized into groups according to the use ever of sulfasalazine (SSZ), hydroxychloroquine (HCQ) or MTX, either as monotherapy or as combinations of these drugs (both sequentially and concurrently in time). The final group consisted of patients who never used any of the three major DMARDs; this resulted in a total of eight groups. These groups were chosen because SSZ, HCQ and MTX are the most commonly used drugs and well represented in our random sample of RA patients.

Logistic regression modeling was used to calculate the odds ratios (ORs) and 95% confidence intervals (95% CIs) of CVD for the various DMARD groups simultaneously. In the regression analysis the group of RA patients who never used SSZ, HCQ or MTX was used as the reference group with a preset OR of 1.00.

The first regression model corrected for age, gender, smoking ever and RA duration. Correcting for age, gender and smoking was done because these variables are known to be associated with CVD but not with the use of certain DMARDs. Correcting for RA duration was done because the chance for a patient to be treated with more than one DMARD increases the longer the duration of the disease. As an additional analysis prednisone use ever was added to this first model.

In the second regression analysis we added the presence of hypertension, diabetes and hypercholesterolemia to the first model. Adding these known risk factors for CVD was done for two reasons. Firstly, because these risk factors could be over- or under-represented in certain DMARD groups and, therefore, falsely influence the cardiovascular risk for these groups. Secondly, correction for known cardiovascular risk factors was done to explore possible pathways by which the investigated DMARDs can influence cardiovascular risk; for example, a DMARD could increase the cardiovascular risk by causing hypertension and this increased risk would disappear after correcting for hypertension.

A third analysis was done using the first model and adding the presence or absence of a positive rheumatoid factor test and erosions on radiographs. This enabled us to calculate the ORs for CVD associated with these two RA related variables.

The three models described above were also used to explore if there was any dose dependency in the possible associations between the DMARD groups and CVD risk. Therefore, we determined the presence of interactions between any of the DMARD groups and the maximum used dosages, days of DMARD use and a cumulative variable. Because the maximum dosages of the different DMARDs are of different quantities (for example, 30 mg for MTX and 3,000 mg for SSZ) we calculated the percentage of the maximum dosages allowed by the Dutch and European medication agencies to be prescribed. For example, 30 mg is the maximum dosage allowed to be prescribed for MTX; therefore, if a patient only used 15 mg at the most, we used 50% as the maximum dosage in the calculations. This way we were able to compare the various DMARD dosages. As a cumulative variable we calculated the maximum percentage of the highest prescribable dosage multiplied by the years this DMARD was used.

A p value of 0.05 or smaller was considered statically significant and all tests were performed using the SPSS 12.0 software package for Windows (SPSS Inc., Chicago, IL, USA).

## Results

### Rheumatoid arthritis patients with and without cardiovascular disease

The baseline characteristics of the RA patients with and without CVD in our study population are shown in Table 1. The RA patients with CVD were significantly older (p < 0.001) and more often male (p = 0.02). Furthermore, they had a longer RA duration (p < 0.001) and were more likely have a positive IgM rheumatoid factor test (p = 0.05) and erosion on radiographs (p = 0.02). The use of DMARDs was also different between the two groups of RA patients. Patients with CVD had a higher median number of used DMARDs (p = 0.01). However, the number of DMARD naive patients and patients who never used SSZ, HCQ or MTX was also higher in the groups of patients with CVD (p = 0.002 and p < 0.001, respectively). Finally, the RA patients with CVD more often had hypertension and hypercholesterolemia (p < 0.001).

### DMARD groups

Various RA and CVD related variables of the entire study population and the different DMARD groups are shown in Table 2. This table also shows the comparison of these variables between a DMARD group and the remainder of the study population. The 'only MTX ever' group had a significantly shorter RA duration (p < 0.001), lower percentage of patients with erosions (p < 0.001) and a higher percentage of diabetics (p = 0.002). The 'only SSZ ever' group showed significantly less erosive patients compared to the remainder of the patients (p = 0.03). The RA duration of the 'only HCQ ever' group was longer than that of the other groups (p = 0.01). The percentage of patients receiving treatment for hypertension was higher in the 'SSZ and HCQ ever' group (p < 0.001). In the 'MTX, SSZ and HCQ ever' group, the RA duration was longer (p = 0.04) and the percentage of erosive patients was higher (p < 0.001).

### Odds ratios for cardiovascular disease

ORs for CVD calculated for three models comparing the various DMARD groups to the RA patients who never used SSZ, HCQ or MTX are presented in Table 3. The first model, correcting for age, gender, smoking ever and RA duration, yielded significant risk reductions for CVD for the 'only MTX ever', the 'MTX and SSZ ever' and 'MTX, SSZ and HCQ ever' groups.

The second model added additional correction for hypertension, diabetes and hypercholesterolemia to the corrections of the first model. This model revealed significant reductions in risk for CVD for the 'MTX and SSZ ever' and 'MTX, SSZ and HCQ ever' groups.

The third model, correcting for the same variables as model 1 plus rheumatoid factor positivity and erosions, showed significant CVD risk reduction for the 'only MTX ever', 'only SSZ ever', 'MTX and SSZ ever' and 'MTX, SSZ and HCQ ever' groups. This third model quantified the ORs for having positive rheumatoid factor test and erosions on radiographs (OR 2.04 (95% CI 1.02 to 4.07) and OR 2.36 (95% CI 0.92 to 6.08), respectively), showing RA patients with poor prognostic signs to have an elevated risk for CVD.

As an additional analysis we added the use ever of prednisone to the first model, giving an OR for CVD of 0.89 (95% CI 0.48 to 1.65), showing no significant association between corticosteroid use and the development of CVD.

### Dose dependency

None of the calculated interactions between the DMARD groups and maximum dosages or the days of DMARD use reached statistical significance (Additional file 1). Moreover, several interactions between the DMARD groups and the cumulative variable did reach statistical significance. In models 1 and 3 we found significant interactions for the 'MTX, SSZ and HCQ ever', the 'MTX and SSZ ever' and the 'SSZ only' groups. In model 2 just the 'MTX, SSZ and HCQ ever' group showed a significant interaction with the cumulative variable. All the observed interactions showed that the cardiovascular risk for these DMARD groups decreased when the cumulative DMARD exposure increased (Additional file 1).

## Discussion

The present study is the first showing a protective role of DMARD use for the risk of cardiovascular morbidity in RA patients. Furthermore, it demonstrates that rheumatoid factor positivity and joint destruction on radiographs both approximately double the risk for CVD.

Earlier studies report on associations between DMARDs and cardiovascular mortality [20,21], the tip of the iceberg, and not on cardiovascular morbidity as the present paper does. Previous literature predominantly focused on MTX and, thereby, ignores the other major conventional DMARDs, such as SSZ and HCQ. Because a substantial number of RA patients use DMARDs other than MTX we chose to include these drugs in our evaluation. Another advantage of the present study is the fact that several CVD- but also RA-related variables were evaluated for their association with CVD.

Mechanisms by which DMARD use could influence the risk for CVD are poorly investigated. HCQ is reported to influence cardiovascular risk by lowering total cholesterol levels [19,22]. Corticosteroids are known to cause insulin resistance, hyperglycemia, weight gain, fluid retention and hypertension, all effects that are associated with an increased cardiovascular risk [23]. The use of MTX can cause a folic acid deficiency with subsequently higher homocysteine levels and, thereby, increasing the risk of CVD [20,24]. On the other hand, Choi and colleagues [21] reported a lower cardiovascular mortality in RA patients using MTX, which was ascribed to the anti-inflammatory quality of MTX.

The reduction of CVD-related morbidity in MTX treated patients is in line with the reduced CVD-related mortality in these patients as found by Choi and colleagues. The results of the present study suggest that the use of other conventional DMARDs, such as SSZ (but not significantly HCQ), is also associated with a reduction in the risk of developing CVD, which strengthens the hypothesis that reducing inflammation is of importance to reduce the risk of CVD. The relationship between inflammation and cardiovascular risk is furthermore underlined by the observation that rheumatoid factor positivity and joint destruction are associated with CVD. These findings stress the importance of aggressive pro-active treatment of RA, as this would not only be beneficial for the outcome of the patients' mobility but could also prevent co-morbidity such as CVD.

There are some limitations to the present study. First, data were obtained by chart review; however, this was done systematically by one observer and classification of CVD was verified in source documents. Second, there is the possibility of 'confounding by indication', that is, more severe disease, in this case indicated by the presence of rheumatoid factor and erosions on radiographs, was associated with a higher risk of CVD; however, patients with these characteristics are also likely to receive more aggressive treatment with DMARDs, which were found to be associated with a cardiovascular protective effect. Therefore, confounding by indication may have biased the results towards null. We can not exclude entirely such a phenomenon; however, the reported associations between DMARDs and CVD risk remained present when variables of severity were included in the analyses.

## Conclusion

RA patients who are being treated with DMARDs, especially MTX, have a reduced risk for CVD in comparison with RA patients who do not use SSZ, HCQ or MTX. We hypothesize that treatment with MTX, and other conventional DMARDs to a smaller extent, is associated with less severe atherosclerosis through suppression of inflammation, which results in a decreased risk for CVD.

## Additional files

Additional file 1 is a series of tables showing dose dependency in DMARD groups and association with CVD; and interaction between DMARD groups with the following variables: percentage maximum dose, days DMARD-use and cumulative dosage years.

## Abbreviations

CVD = cardiovascular disease; DMARD = disease-modifying antirheumatic drug; HCQ = hydroxychloroquine; MTX = methotrexate; OR = odds ratio; RA = rheumatoid arthritis; SSZ = sulfasalazine; CI = confidence interval.

## Competing interests

The authors declare that they have no competing interests.

## Authors' contributions

VvH was responsible for the conception and design of the present study, data acquisition, analysis and interpretation and was involved in drafting the manuscript. MN was involved in the present study's conception, the interpretation of the data and revising the manuscript critically. JT was responsible for the study design and interpretation of the data and was involved in writing the manuscript, focusing on the statistical analyses. BD was involved in the design of the present study and gave his intellectual input during the drafting process. AV was also involved in the conception and design of the study, interpretation of the data and coordination of the drafting of the manuscript. All authors read and approved the final manuscript.

## Supplementary Material

Additional file 1 Series of tables showing dose dependency in DMARD groups and association with CVD; and interaction between DMARD groups with the following variables: percentage maximum dose, days DMARD-use and cumulative dosage years. Click here for file

## Figures and Tables

**Table 1:** Characteristics of rheumatoid arthritis patients with and without cardiovascular disease

	RA without CVD (*n *= 541)	RA with CVD (*n *= 72)	p value
Demographic variables			
Mean age, years (SD)	62 (11)	67 (10)	<0.001^a^
Percentage females	72	58	0.02^a^
RA related variables			
Median disease duration, years (IQ range)	7.7 (5–11)	10.6 (8–13)	<0.001^a^
Percentage IgM-RF positive	70	82	0.05^a^
Percentage erosive patients	80	92	0.02^a^
Median number of used DMARDs (IQ range)	2 (2–3)	3 (1–3)	0.01^a^
Percentage DMARD naive patients	3	10	0.002^a^
Percentage never SSZ, HCQ or MTX	5	17	<0.001^a^
Percentage SSZ ever	78	65	0.02^a^
Percentage HCQ ever	40	38	0.67
Percentage MTX ever	72	44	<0.001^a^
Percentage prednisone ever	31	25	0.32
CVD related variables			
Percentage smoking ever	64	65	0.83
Percentage hypertension	19	49	<0.001^a^
Percentage diabetes	5	10	0.14
Percentage hypercholesterolemia	2	21	<0.001^a^

Comparison made using Students' t-tests or Mann-Whitney U tests for the continues variables and Pearson's Chi-square tests for dichotomic variables. ^a^Significant. CVD, cardiovascular disease; DMARD, disease modifying anti-rheumatic drug; HCQ, hydroxychloroquine; IQ range, inter-quartile-range; MTX, methotrexate; RA, rheumatoid arthritis; RF, rheumatoid factor; SD, standard deviation; SSZ, sulfasalazine.

**Table 2:** Rheumatoid arthritis and cardiovascular disease related variables per DMARD-group including associated p values

Groups	n (percent)	RA related variables (p value)			CVD related risk factors (p value)		
		
		RA duration	Percentage RF	Percentage erosive	Percentage hypertension	Percentage diabetes	Percentage hypercholesterolemia
Entire group	613 (100)	9	72	82	22	6	4
Never MTX, SSZ or HCQ	37 (6)	12 (0.25)	68 (0.58)	70 (0.06)	24 (0.74)	11 (0.19)	10 (0.11)
Only MTX ever	51 (8)	5 (<0.001)^a^	61 (0.09)	57 (<0.001)^a^	12 (0.06)	16 (0.002)^a^	6 (0.77)
Only SSZ ever	82 (13)	8 (0.29)	70 (0.65)	73 (0.03)^a^	26 (0.38)	2 (0.16)	9 (0.14)
Only HCQ ever	36 (6)	12 (0.01)^a^	70 (0.77)	81 (0.85)	17 (0.46)	3 (0.42)	4 (0.96)
MTX and SSZ ever	199 (33)	9 (0.10)	75 (0.18)	85 (0.12)	22 (0.97)	6 (0.80)	1 (0.11)
MTX and HCQ ever	20 (3)	10 (0.83)	75 (0.73)	80 (0.12)	22 (0.97)	6 (0.80)	1 (0.11)
SSZ and HCQ ever	39 (7)	10 (0.39)	69 (0.74)	90 (0.20)	39 (<0.001)^a^	3 (0.36)	0 (0.35)
MTX, SSZ and HCQ ever	149 (24)	10 (0.04)^a^	73 (0.63)	91 (<0.001)^a^	20 (0.56)	5 (0.48)	5 (0.90)

Comparison calculated using a Students' t-tests or Pearson's Chi-square tests, relative to the remainder of the population. ^a^Significant. 'RA duration' is in years; 'Percentage RF' refers to positive test for IgM rheumatoid factor; 'Percentage erosive' refers to erosions on radiographs of hands and/or feet. CVD, cardiovascular disease; DMARD, disease modifying anti-rheumatic drug; HCQ, hydroxychloroquine; MTX, methotrexate; RA, rheumatoid arthritis; RF, rheumatoid factor; SSZ, sulfasalazine

**Table 3:** Odds ratios for cardiovascular disease

Groups	Model 1 OR (95 percent CI)	Model 2 OR (95 percent CI)	Model 3 OR (95 percent CI)
Never MTX, SSZ or HCQ (reference)	1.0	1.0	1.0
Only MTX ever	0.16 (0.04–0.66)^a^	0.47 (0.07–3.23)	0.11 (0.02–0.56)^a^
Only SSZ ever	0.42 (0.16–1.10)	0.31 (0.07–1.33)	0.37 (0.14–0.99)^a^
Only HCQ ever	0.55 (0.18–1.67)	0.45 (0.10–2.04)	0.47 (0.15–1.46)
MTX and SSZ ever	0.20 (0.08–0.51)^a^	0.24 (0.07–0.85)^a^	0.16 (0.06–0.42)^a^
MTX and HCQ ever	0.22 (0.04–1.19)	0.54 (0.08–3.66)	0.19 (0.04–1.02)
SSZ and HCQ ever	0.44 (0.14–1.41)	0.34 (0.05–2.16)	0.37 (0.11–1.24)
MTX, SSZ and HCQ ever	0.20 (0.08–0.54)^a^	0.27 (0.07–0.99)^a^	0.16 (0.06–0.43)^a^

Model 1: correcting for age, gender, smoking and rheumatoid arthritis duration. Model 2: identical to 'Model 1' plus correction for hypertension, diabetes and hypercholesterolemia. Model 3: identical to 'Model 1' plus correction for a positive rheumatoid factor test and erosions. ^a^Significant. CI, confidence interval; HCQ, hydroxychloroquine; MTX, methotrexate; OR, odds ratio; SSZ, sulfasalazine.
