# Supplementary material for: Disease-modifying antirheumatic drugs are associated with a reduced risk for cardiovascular disease in patients with rheumatoid arthritis: a case control study
Source: Arthritis Res Ther. 2006 Sep 20;8(5):R151. doi: 10.1186/ar2045 (PMC1779436; doi:10.1186/ar2045)
Supplement: Additional file 1 — Series of tables showing dose dependency in DMARD groups and association with CVD; and interaction between DMARD groups with the following variables: percentage maximum dose, days DMARD-use and cumulative dosage years. [file ar2045-S1.doc]

MODEL 1

MODEL 2

MODEL 3
